# CD47 overexpression is related to tumour‐associated macrophage infiltration and diffuse large B‐cell lymphoma progression

**DOI:** 10.1002/ctm2.1532

**Published:** 2024-01-09

**Authors:** Yi‐Ge Shen, Meng‐Meng Ji, Hong‐Mei Yi, Rong Shen, Di Fu, Shu Cheng, Chuan‐Xin Huang, Li Wang, Peng‐Peng Xu, Hong‐Jing Dou, Wei‐Li Zhao

**Affiliations:** ^1^ Shanghai Institute of Hematology State Key Laboratory of Medical Genomics National Research Center for Translational Medicine at Shanghai Ruijin Hospital Affiliated to Shanghai Jiao Tong University School of Medicine Shanghai China; ^2^ Department of Pathology Ruijin Hospital Affiliated to Shanghai Jiao Tong University School of Medicine Shanghai China; ^3^ Department of Immunobiology and Microbiology Shanghai Institute of Immunology Shanghai Jiao Tong University School of Medicine Shanghai China; ^4^ Pôle de Recherches Sino‐Français en Science du Vivant et Génomique Laboratory of Molecular Pathology Shanghai China; ^5^ State Key Laboratory of Metal Matrix Composites School of Materials Science and Engineering National Research Center for Translational Medicine at Shanghai Shanghai Jiao Tong University Shanghai China

Dear Editor,

Cluster of differentiation 47 (CD47) belongs to the immunoglobulin superfamily and is considered an adverse prognostic risk factor for diffuse large B‐cell lymphoma (DLBCL) patients.^1^ Accumulating evidence reveals that CD47 functions as a promising therapeutic target of lymphoma immunotherapy.^1^ Aiming to establish more effective targeted approaches, we performed systematic analyses of genomic and transcriptomics to investigate the association between genetic mutations and tumour microenvironment with CD47 overexpression in DLBCL. Among 702 newly diagnosed DLBCL patients (Figure S1), 460 patients (65.5%) were classified into a high CD47 expression group according to the survival receiver operating characteristic (ROC) curve. High CD47 expression was significantly associated with elevated serum lactic dehydrogenase (65.0% or 299/460 vs. 57.4% or 139/242; *p* = .0493) and increased proportion of non‐germinal centre B‐cell subtype (66.1% or 271/410 vs. 52.9% or 111/210; *p* = .0013) (Table S1). To evaluate the predictive value of CD47 overexpression on disease outcomes, we compared the prognosis of 436 patients with rituximab, cyclophosphamide, doxorubicin, vincristine, and prednisone (R‐CHOP) treatment. Patients with high CD47 expression showed a remarkable decrease in 4‐year progression‐free survival (PFS, 56.5% vs. 69.0%; *p* = .0032) and overall survival (OS, 73.4% vs. 86.1%; *p* = .0017) rate relative to those with low CD47 expression (Figure 1A,B), with the median follow‐up time of 35.6 months. Furthermore, multivariate analysis confirmed CD47 as an independent factor of worse prognosis (PFS, *p* = .0232; OS, *p* = .0044, Figure 1C,D).

**FIGURE 1:**
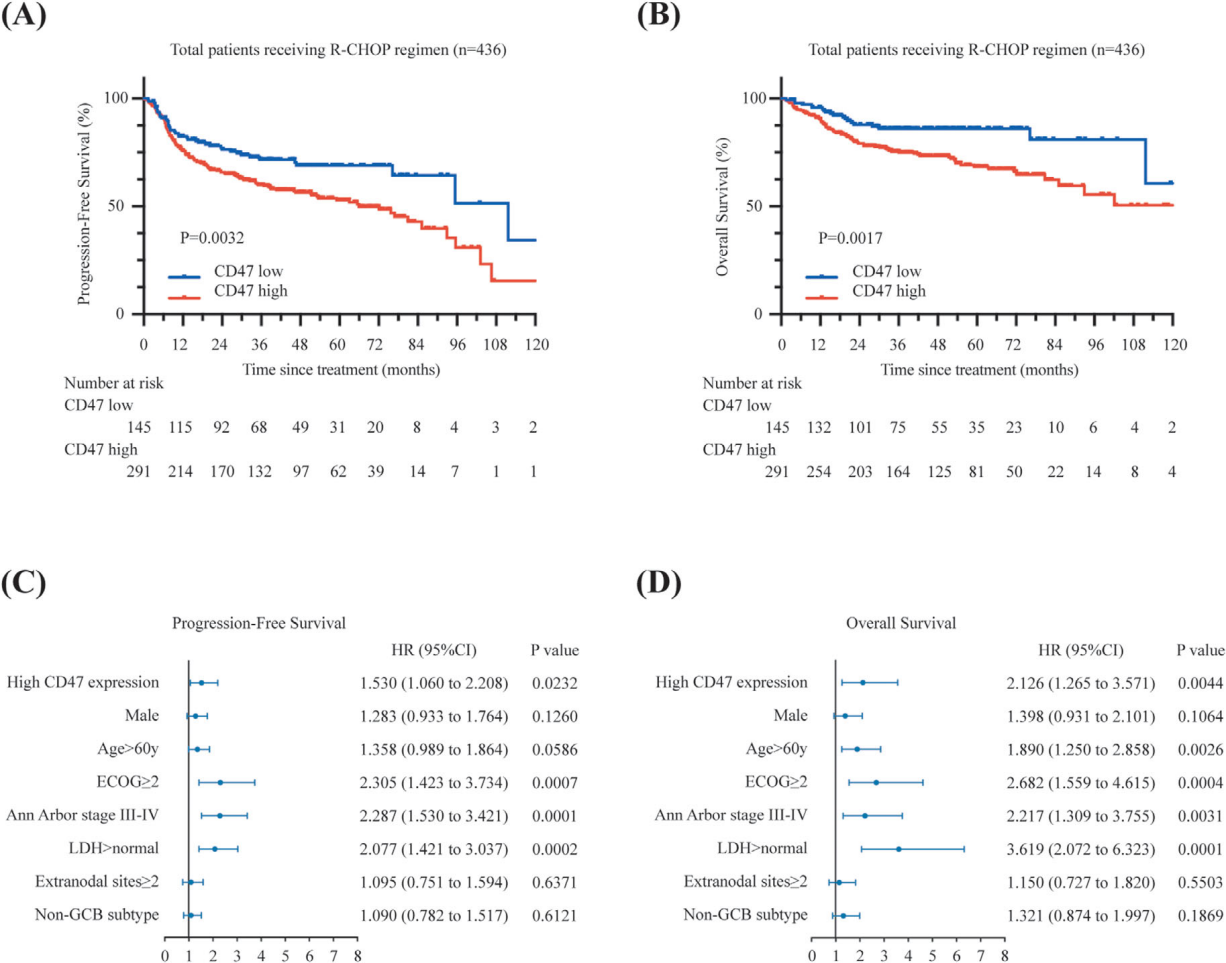
Clinical outcomes according to CD47 expression in diffuse large B‐cell lymphoma (DLBCL) patients with rituximab, cyclophosphamide, doxorubicin, vincristine, and prednisone (R‐CHOP) regimen. (A and B) Kaplan–Meier curves of progression‐free survival (PFS) and overall survival (OS) according to CD47 mRNA expression in DLBCL patients treated with R‐CHOP regimen. Data are analysed by log‐rank test. (C and D) Univariate analysis of predictors for PFS and OS. Hazard ratios (HR), 95% confidence intervals (95% CIs) and *p*‐values are indicated on the right of each forest plot.

Based on whole exome/genome sequencing and targeted sequencing data, we described a comprehensive analysis of the genomic alteration landscape of DLBCL with CD47 overexpression. Among oncogenic mutations, *TBL1XR1* (11.3% or 52/460 vs. 5.0% or 12/242; *p* = .0055), *NOTCH1* (7.2% or 33/460 vs. 2.9% or 7/242; *p* = .0200) and *PRDM1* (10.2% or 47/460 vs. 5.8% or 14/242; *p* = .0475) mutations were significantly increased in patients with high CD47 expression (Figure 2A), while *SOCS1* (11.5% or 53/460 vs. 18.2% or 44/242; *p* = .0151) and *HIST1H1C* (4.8% or 22/460 vs. 9.1% or 22/242; *p* = .0252) mutations were significantly increased in patients with low CD47 expression. Gene set enrichment analysis further revealed that CD47 overexpression was remarkably linked to enriched oncogenic pathways, including mitogen‐activated protein kinase signaling pathway (MAPK), Phosphatidylinositol3 kinase‐protein kinase B signaling pathway (PI3K–AKT) and RAS signaling pathway (RAS). Immune regulation pathways, including cytokine interaction, protein kinase A signaling pathway (cAMP) and calcium signalling pathways, were also upregulated (Figure 2B). As for the molecular classification of DLBCL, a higher proportion of one type of molecular subtype with high frequency of MYD88 and CD79B mutations (MCD)‐like (62/393 or 15.8% vs. 19/222 or 8.6%; *p* = .0110) and N1‐like (21/393 or 5.3% vs. 4/222 or 1.8%; *p* = .0327) genotypes was observed in patients with high CD47 expression, while ST2‐like genotype (22/393 or 5.6% vs. 23/222 or 10.4%; *p* = .0294) was more frequently observed in those with low CD47 expression (Figure 2C). These findings supported that CD47 was involved in genetic alterations and pathway dysregulation, rending potential target signalling cascades for treating high CD47 DLBCL. Encouraged by the synergistic effects of bispecific antibodies targeting CD47 and other haematological surface markers,^2^ correlation analysis of RNA sequencing data also showed that CD47 was significantly associated with CD19, CD20 and 4‐1BB expression (Figure 2D). Bispecific antibodies, such as CD19/CD47, CD20/CD47, 4‐1BB/CD47 and PD‐L1/CD47 may show stronger anti‐tumour effects than CD47 monoclonal antibodies in both pre‐clinical and clinical researches. These observations indicated promising links of CD47 with other haematological surface markers as alternative approaches to CD47‐targeted immunotherapy in DLBCL.

**FIGURE 2:**
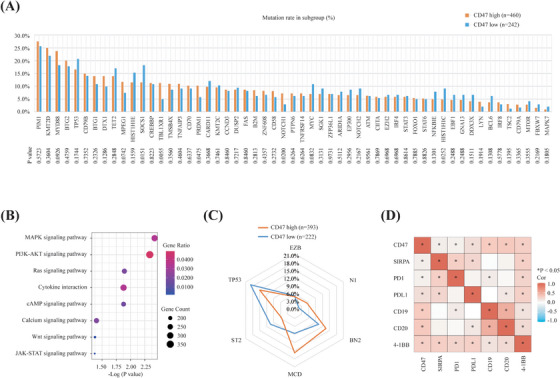
Relationship between oncogenic mutations and CD47 expression in diffuse large B‐cell lymphoma (DLBCL). (A) Prevalence of genetic mutations in patients with high CD47 expression (*n* = 460) and low CD47 expression (*n* = 242) of DLBCL. The lower graph indicates *p*‐values comparing different prevalence in two groups. (B) Upregulated gene ontology (GO) terms in high CD47 expression (*n* = 460), as compared to low CD47 expression (*n* = 242). The colour of points indicates gene ratio of dysregulated pathways in the two groups. The size of points indicates number of genes included in each gene set. (C) Prevalence of DLBCL subtypes classified by LymphPlex method in patients with high CD47 expression (*n* = 393) and low CD47 expression (*n* = 222). (D) Correlations of CD47 mRNA expression with other immune checkpoints in DLBCL. Data are analysed by Spearman's correlation test. Asterisks mark all bivariate correlations that are significant (*p* < .05).

Signal regulatory protein α (SIRPα) and thrombospondin‐1 (TSP1) are both important ligands binding to CD47. According to the literature, the interaction of CD47 and TSP1 suppresses the binding of SIRPα and regulates nitric oxide signalling in vascular cells.^3^, ^4^ Indeed, the TSP1–CD47–SIRPα interactome plays an important role in immune suppression. In our study, we found that mRNA expression of SIRPα was upregulated in the high group, while TSP1 showed no difference between the two groups (Figure S2). Engagement of CD47 by SIRPα in tumours induces a downregulatory signal that suppresses the phagocytic function of macrophages and thereby represents a ‘don't‐eat‐me’ signal contributing to tumour growth and progression.^5^ Besides, upregulation of CD47 facilitates tumour immune escape by influencing the polarisation of macrophages.^6^ Therefore, considering the important roles of CD47 in regulating macrophage function, tumour immunophenotyping method was performed using RNA sequencing data to compare the infiltration of macrophage subsets. Our results revealed higher infiltration of M2‐polarised macrophages in patients with high CD47 expression (*p* = .0050), characterised by increased cell surface molecules such as CD200R1, TGFBR1 and CD226 (Figure 3A,B). Consistently, key chemokines and cytokines involved in M2‐polarised macrophages recruitment activity,^7^ including a type of chemokine also called monocyte chemoattractant protein‐1 (CCL2), chemokine ligand 16 (CXCL16), cytokine interleukin‐10 (IL‐10) and cytokine transforming growth factor‐β (TGF‐β), were significantly upregulated in high CD47 DLBCL, as compared to low CD47 DLBCL (all *p* < .0001; Figure 3D,E). Previous studies showed that the infiltration of M2‐polarised macrophages leads to chemotherapy resistance and inferior prognosis of DLBCL,^8^ which corroborated our findings. MAPK and PI3K–AKT pathways are also involved in M2‐polarised macrophage differentiation, proliferation and activity,^9^ which reveal downstream signalling of CD47 in regulating macrophages. Moreover, CD47 overexpression was related to inflammatory lymphoma microenvironment (LME‐IN) (152/460 or 33.0% vs. 29/242 or 12.0%; *p* < .0001; Figure 3C) subtype, which presented decreased anti‐tumour immunity, such as enrichment of immunosuppressive and pro‐lymphoma cytokines.^10^ Mesenchymal LME subtype was more frequently observed in those with low CD47 expression (121/460 or 26.3% vs. 106/242 or 43.8%; *p* < .0001). Together, CD47 could promote M2 polarisation of macrophages and provoke an immunosuppressive microenvironment. Further explorations of the underlying influence on the DLBCL microenvironment are needed for optimising anti‐cancer strategies to improve the outcomes of CD47‐overexpressing DLBCL.

**FIGURE 3:**
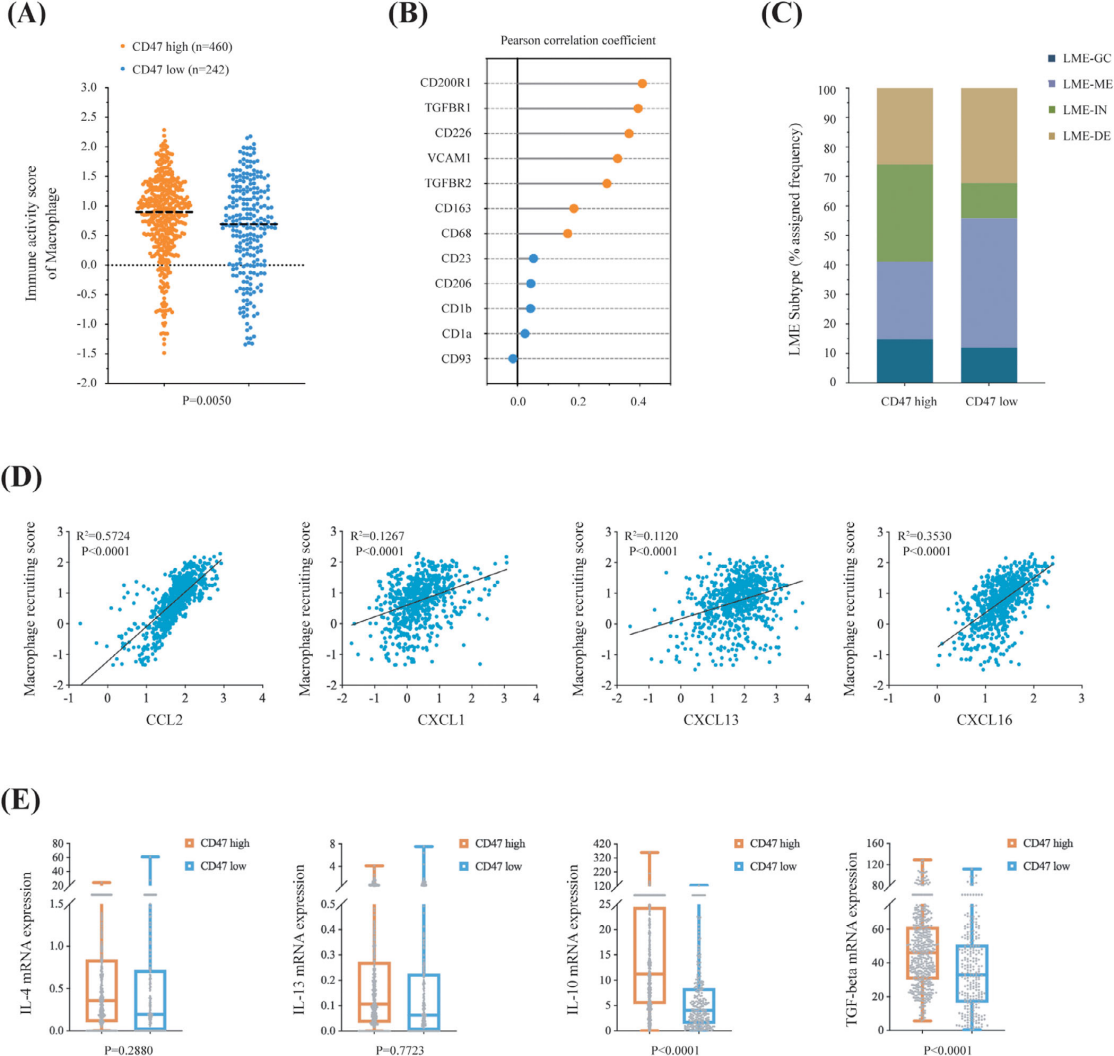
Relationship between intratumour immune cells and CD47 expression in diffuse large B‐cell lymphoma (DLBCL). (A) Immunity activity scores of macrophages in patients with high CD47 expression (*n* = 460) and low CD47 expression (*n* = 242). The lower graph indicates *p*‐values comparing different scores in two groups. (B) Correlations between the expression of M2‐polarised macrophage surface molecules and CD47. Orange colour marks all bivariate correlations that are significant (*p* < .05). (C) Prevalence of DLBCL subtypes classified by lymphoma microenvironment (LME) categories. (D) Correlations between the expression of chemokines and the abundance of macrophages. *p*‐Values and *R* ^2^ values are indicated in each plot. (E) Relative proportion of M2‐polarised cytokines expression between high CD47 expression (*n* = 460) and low CD47 expression (*n* = 242). Data are presented as median and range. *p*‐Values comparing different prevalence in two groups are indicated above the columns.

Recent studies have adopted certain criteria to evaluate CD47 positivity using immunohistochemistry (IHC) analysis, while the cut‐off value for CD47 expression has not been determined in DLBCL. The expression of CD47 was evaluated with four levels according to intensity (Figure 4A). CD47 expression was found in 180 of 212 (84.9%) patient samples with the median number of positive tumour cell rate at 50% (Figure 4B). By performing the ROC curve between CD47 IHC and mRNA expression, we found the threshold of stained tumour cells >60% as IHC high group (Figure 4C). The area under the curve was obtained as .8134 (95% confidence interval .7417–.8851) with statistical significance. This cut‐off point was validated by observation of significant survival differences between the two different protein expression groups (Figure 4D,E), which could predict the clinical adaptive population of CD47 blockade.

**FIGURE 4:**
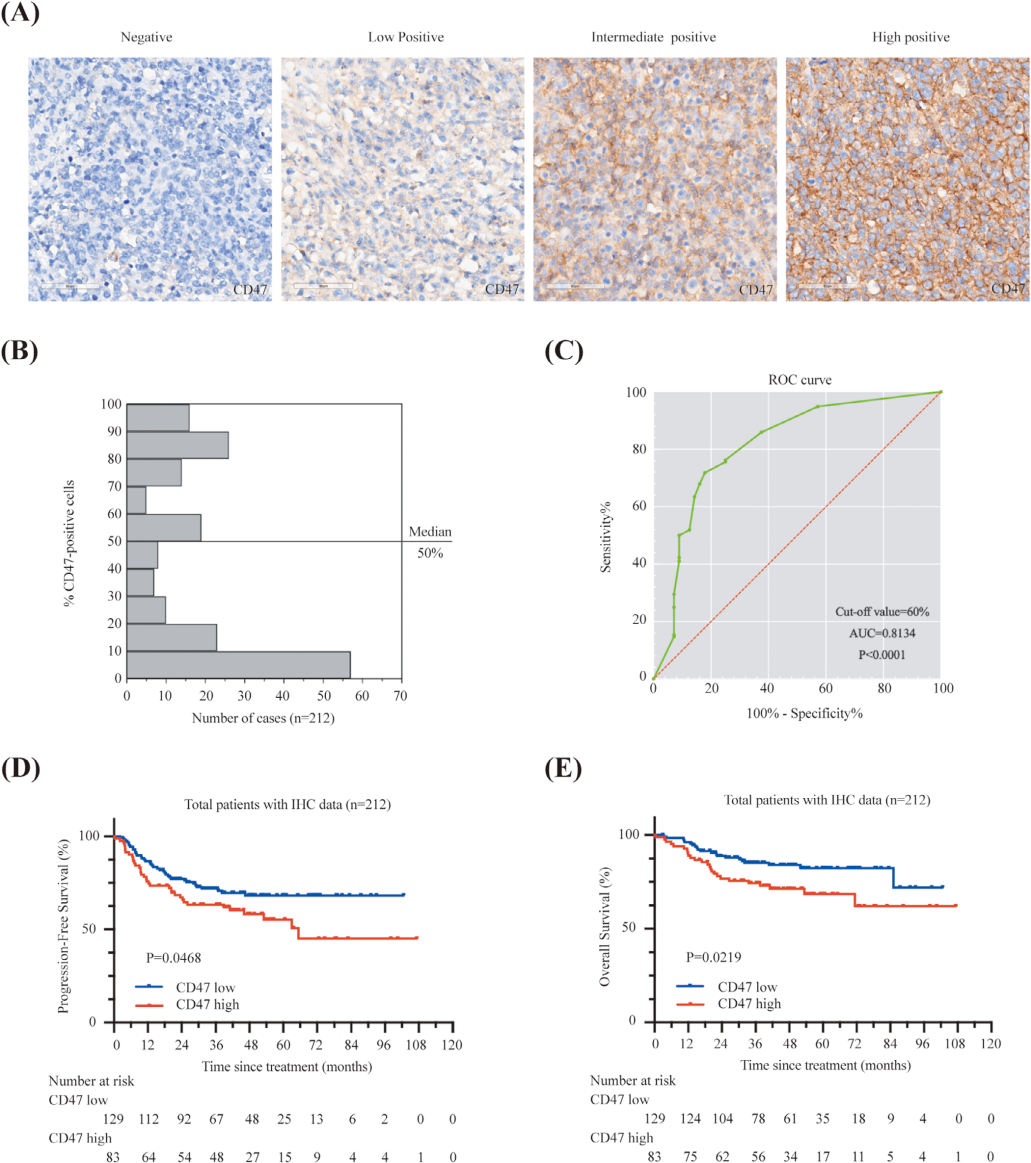
Immunohistochemical analysis of CD47 expression in diffuse large B‐cell lymphoma (DLBCL). (A) Immunohistochemical (IHC) images and positivity of CD47 expression on patient tumour samples. Original magnification, ×400 for all panels. (B) Distributions of the rate of CD47‐positive tumour cells. (C) Optimal cut‐off estimated by receiver operating characteristic (ROC) curve analysis. The area under the curve (AUC) was indicated in the plot. (D and E) Kaplan–Meier curves of progression‐free survival (PFS) and overall survival (OS) according to CD47 IHC expression in DLBCL patients treated with rituximab, cyclophosphamide, doxorubicin, vincristine, and prednisone (R‐CHOP) regimen. Data are analysed by log‐rank test.

In conclusion, CD47 is an adverse prognostic biomarker, linking to genomic alterations and immunosuppressive microenvironment in DLBCL. A better understanding of the clinical and biological significance of CD47 may be helpful in selecting potential immunotherapeutic targets for future mechanism‐based treatment in DLBCL.

## AUTHOR CONTRIBUTIONS

Wei‐Li Zhao designed and supervised the study. Meng‐Meng Ji and Yi‐Ge Shen collected the data and performed the analyses. Hong‐Mei Yi and Chuan‐Xin Huang reviewed the histopathologic diagnoses and gave technical support. Rong Shen and Di Fu prepared biological samples. Shu Cheng, Li Wang and Hong‐Jing Dou provided executive support and data surveillance. Peng‐Peng Xu and Wei‐Li Zhao verified the underlying data. Yi‐Ge Shen and Wei‐Li Zhao interpreted the results and wrote the manuscript. All authors had access to the study data, made the final decision on where to publish and contributed to the preparation, review and approval of the manuscript.

## CONFLICT OF INTEREST STATEMENT

The authors declare they have no conflicts of interest.

## ETHICS STATEMENT

The study was approved by the Shanghai Ruijin Hospital Review Board. Informed consent was obtained from all patients in accordance with the Declaration of Helsinki. All tissues used for immunohistochemistry were obtained from Shanghai Ruijin Hospital with written informed consent. The study was approved by the Ethics Committees of Shanghai Ruijin Hospital. All experimental procedures followed the rules of the Committee on Animal Care of Shanghai, China.

## CONSENT FOR PUBLICATION

All authors have agreed to publish this manuscript.

## Supporting information

Supporting Information

## Data Availability

All the methods or reagents we used are accessible on the market. Genomic and gene expression data have been deposited on https://www.biosino.org/node in project OEP001143. Proposals requesting individual participant data that underlie the results reported in this paper (after de‐identification) can be sent to zhao.weili@yahoo.com.
