# Supplementary material for: CD47 overexpression is related to tumour‐associated macrophage infiltration and diffuse large B‐cell lymphoma progression
Source: Clin Transl Med. 2024 Jan 9;14(1):e1532. doi: 10.1002/ctm2.1532 (PMC10775178; doi:10.1002/ctm2.1532)

**CD47 overexpression is related to tumor-associated macrophage infiltration and diffuse large B-cell lymphoma progression**

Yi-Ge Shen ^1†^, Meng-Meng Ji ^1†^, Hong-Mei Yi ^2†^, Rong Shen ^1^, Di Fu ^1^, Shu Cheng ^1^, Chuan-Xin Huang ^3^, Li Wang ^1,4^, Peng-Peng Xu ^1^, Hong-Jing Dou ^5^, Wei-Li Zhao ^1,4^*****

^1^ Shanghai Institute of Hematology, State Key Laboratory of Medical Genomics; National Research Center for Translational Medicine at Shanghai, Ruijin Hospital Affiliated to Shanghai Jiao Tong University School of Medicine, Shanghai, China

^2^ Department of Pathology, Ruijin Hospital Affiliated to Shanghai Jiao Tong University School of Medicine, Shanghai, China

^3^ Department of Immunobiology and Microbiology, Shanghai Institute of Immunology, Shanghai Jiao Tong University School of Medicine, Shanghai, China

^4^ Pôle de Recherches Sino-Français en Science du Vivant et Génomique, Laboratory of Molecular Pathology, Shanghai, China

^5^ State Key Laboratory of Metal Matrix Composites, School of Materials Science and Engineering, National Research Center for Translational Medicine at Shanghai, Shanghai Jiao Tong University, Shanghai, China

***Correspondence**: Wei-Li Zhao, Email: zhao.weili@yahoo.com, Shanghai Institute of Hematology; State Key Laboratory of Medical Genomics; National Research Center for Translational Medicine at Shanghai; Ruijin Hospital Affiliated to Shanghai Jiao Tong University School of Medicine, 197 Ruijin Er Road, Shanghai 200025, China, Tel: 0086-21-64370045, Fax: 0086-21-64743206.

**†** These authors equally contributed to this work.

**Supplementary Materials and Methods**

**Patients and samples**

A flow chart is outlined in Figure S1 to describe the patient selection and methods. We investigated 702 patients with newly diagnosed DLBCL, including 22 cases of double-hit lymphoma, from July 2000 to December 2021 based on registry data, with the last follow-up through May 31, 2023. All cases were reviewed and confirmed by pathologists and diagnosed in accordance with the WHO classification.^1^ Patients of primary central nervous system lymphoma, primary mediastinal lymphoma, or primary cutaneous lymphoma were excluded. Clinical data included age, gender, International Prognostic Index (IPI), Eastern Cooperative Oncology Group (ECOG) performance status, Ann Arbor stage, serum lactate dehydrogenase (LDH), extranodal involvement, and cell of origin. Survival analysis was performed on 436 patients who received the R-CHOP regimen, excluding other chemotherapy, radiotherapy, surgery alone, or died before treatment (n=266). Available tumor samples of 702 cases were performed DNA and RNA sequencing, respectively, for detection of genetic aberrations, gene set enrichment analysis (GSEA), and tumor immunophenotyping (TIP). The study was approved by the Shanghai Ruijin Hospital Review Board, and informed consent was obtained in accordance with the Declaration of Helsinki.

**DNA sequencing**

Genomic DNA was extracted from frozen tumor tissue by a QIAamp DNA Mini Kit (Qiagen, Hilden, Germany), or from formalin-fixed paraffin-embedded (FFPE) tumor tissue by a GeneRead DNA FFPE Tissue Kit (Qiagen), based on the manufacturer’s guidelines. Whole-exome sequencing (WES) was carried out on frozen tumor tissue or FFPE tumor tissue quality controlled by agarose gel electrophoresis for 117 patients. Whole-genome sequencing (WGS) was performed on frozen tumor tissue for 193 patients. For targeted sequencing of 55 lymphoma-associated genes,^2^ 392 patients with FFPE tumor tissue quality-controlled by agarose electrophoresis were included. Genome Analysis Toolkit (GATK, v3.7.0), Haplotype Caller, and GATK Unified Genotyper were applied to call single nucleotide variations (SNVs) and indels, which were mapped to the genome location using the UCSC Genome Browser (http://genome.ucsc.edu). The Refseq database (Human Reference Genome version hg19) was used as the reference genome. The filtration of detected SNVs and indels was performed by homemade pipeline with the above software. Detailed procedures for DNA sequencing were carried out as previously reported.^2^

**RNA sequencing**

Total RNA was extracted with Trizol and RNeasy Mini kit (Qiagen) using frozen tumor tissue samples. TruSeq RNA Samples Preparation Kit (Illumina) was used to construct the sequencing library. Qubit (Thermo Fisher Scientific) and Agilent BioAnalyzer 2100 (Agilent) were used to quantify the concentration of the resulting sequencing libraries and the size distribution. Read pairs were aligned to RefSeq hg19 by Burrows-Wheeler Aligner version 0.7.13-r1126. Transcript counts table files were generated by the HTSeq.^3^ Bioinformatic analyses were performed by r 3.5.1, using R package “sva” to remove the batch effect. Raw reads were normalized, and differentially expressed genes were obtained with R package “limma” (v3⋅38⋅3). Detailed procedures for RNA sequencing were performed as previously reported.^2^

**Bioinformatic analyses**

Bioinformatic analyses were performed by r 3.6.1, using R package “sva” to remove the batch effect. The metric method used to rank the genes was Signal2Noise by default. Differentially expressed genes (DEGs) were obtained by R package ‘limma’ (v3.40.6) with an adjusted *P* value of 0.05 and fold change of ≥ 2 or ≤ -2. GSEA was presented as the upregulation or downregulation of the desired gene set using GSEA v4.0.1 software (http://software.broadinstitute.org/gsea/downloads.jsp) with MSigDB (Molecular Signatures Database) curated gene sets (c2.cp.kegg.v6.2.symbols.gmt) and (c5.all.v7.1.symbols.gmt).^4,5^ Pathways were considered statistically significant when the *P* value was <0.05, and the false discovery rate was <0.25.

**Tumor immunophenotyping**

For large-scale characterization of tumor cellular heterogeneity, cell type enrichment scores were generated using tracking tumor immunophenotyping (TIP, http://biocc.hrbmu.edu.cn/TIP21) method, which contains 178 signature genes and 23 signature gene sets involved in the cancer-immunity cycle as described in published studies.^6^ With the RNA sequencing data, the activity scores based on their stimulatory or inhibitory role in the anti-tumor immune response were calculated by single-sample GSEA of all signatures corresponding to the cell type. Using simulations of gene expression for each cell type, the non-linear association between the scores to a linear scale was transformed and the dependencies between cell type scores were derived. The final score of each signature gene set for each sample was adjusted by examining the difference between the normalized scores of the stimulatory gene set and the inhibitory gene set.

**Molecular classification**

Based on the Hans algorithm, all cases were dichotomized to germinal-center B-cell-like (GCB) subtype or non-GCB subtype, with 30% cutoff value of CD10, BCL6, and MUM1.^7^ A simplified 38-gene algorithm LymphPlex based on the information on mutations of 35 genes and rearrangements of three genes (BCL2, BCL6, and MYC) was designed, as previously reported.^8^ By applying this method, 615 patients with DNA sequencing data were assigned into one of the following genetic subtypes: TP53^mut^, MCD-like, BN2-like, N1-like, EZB-like (including EZB-like MYC- and EZB-like MYC+), ST2-like, or not otherwise specified (NOS).

**Lymphoma microenvironment classification**

Based on the lymphoma microenvironment (LME) categories as described by a previous study,^9^ DLBCL subtypes revealed by LME signatures were identified using LME categories. Gene-expression profiles in 702 patients were analyzed, calculated, and integrated, to define the diversity in the composition and functionality of DLBCL microenvironments. Identified patients were classified into four LME categories including germinal center-like (GC), mesenchymal (MS), inflammatory (IN), and depleted (DP) according to the classification tool.

**Immunohistochemistry**

Immunohistochemistry (IHC) was performed on 5 μm paraffin sections using the indirect immunoperoxidase method by antibodies against CD47. Anti-CD47 monoclonal antibody (ab218810, diluted 1:2000; Abcam) was used as the primary antibody. For assessment of CD47 expression, the stained paraffin sections were evaluated at 400×magnification. CD47 was primarily localized on the membrane of tumor cells. Protein expression levels were recorded according to the percentage of stained tumor cells in a representative high-power field. Any staining in lymphatic, lymphocytic, or endothelial tissue was discounted. In the case of multiple staining populations, the most intensely staining subpopulation defined the expression level. The recording was performed in a completely blinded manner and, if there was a conflict, was scored unanimously by two reviewers.

**Statistical analysis**

The cut-off value of RNA sequencing expression was obtained by the survival receiver operating characteristic (ROC) curve. Clinical characteristics of patients were ascertained using Pearson’s χ2 test or Fisher’s exact test. Progression-free survival (PFS) was calculated from the date of diagnosis to the date when disease progression was identified or the date of last follow-up. The endpoint of overall survival (OS) was defined as death from any cause. Kaplan-Meier method was used to carry out survival functions and the Log-rank test indicated significant differences. Univariate and multivariate Cox models were applied to evaluate the proposed risk factors. All *P* values of multiple comparisons were adjusted using the FDR method.^10^ Results P<0.05 were considered significant. Oncogenic mutations, genetic subtypes, and four LME categories were analyzed by Chi-square test. Independent t-test and Spearman correlation analysis were used to analyze the differences in immunity activity scores and cytokines expression in the two groups. Statistical analyses were performed by Statistical Package for the Social Sciences (SPSS) 26.0 software (SPSS Inc., Chicago, IL).

**Study approval**

The study was approved by the Shanghai Ruijin Hospital Review Board. Informed consent was obtained from all patients in accordance with the Declaration of Helsinki. All tissues used for immunohistochemistry were obtained from Shanghai Ruijin Hospital with written informed consent. The study was approved by the Ethics Committees of Shanghai Ruijin Hospital. All experimental procedures followed the rules of the Committee on Animal Care of Shanghai, China.

**Experimental blind method**

No blinding was used in data collection and analysis.

**Statement**

Artificial intelligence (AI) was not involved in manuscript preparation.

**REFERENCES**

1. Swerdlow SH, Campo E, Pileri SA, et al. The 2016 revision of the World Health Organization classification of lymphoid neoplasms. Blood. 2016;127(20):2375-2390.
2. Shen R, Xu PP, Wang N, et al. Influence of oncogenic mutations and tumor microenvironment alterations on extranodal invasion in diffuse large B-cell lymphoma. Clin Transl Med. 2020;10(7):e221.
3. Anders S, Pyl PT, Huber W. HTSeq--a Python framework to work with high-throughput sequencing data. Bioinformatics. 2015;31(2):166-169.
4. Hänzelmann S, Castelo R, Guinney J. GSVA: gene set variation analysis for microarray and RNA-seq data. BMC Bioinformatics. 2013;14:7.
5. Subramanian A, Tamayo P, Mootha VK, et al. Gene set enrichment analysis: a knowledge-based approach for interpreting genome-wide expression profiles. Proc Natl Acad Sci U S A. 2005;102(43):15545-15550.
6. Xu L, Deng C, Pang B, et al. TIP: A Web Server for Resolving Tumor Immunophenotype Profiling. Cancer Res. 2018;78(23):6575-6580.
7. Hans CP, Weisenburger DD, Greiner TC, et al. Confirmation of the molecular classification of diffuse large B-cell lymphoma by immunohistochemistry using a tissue microarray. Blood. 2004;103(1):275-82.
8. Shen R, Fu D, Dong L, et al. Simplified algorithm for genetic subtyping in diffuse large B-cell lymphoma. Signal Transduct Target Ther. 2023;8(1):145.
9. Kotlov N, Bagaev A, Revuelta MV, et al. Clinical and Biological Subtypes of B-cell Lymphoma Revealed by Microenvironmental Signatures. Cancer Discov. 2021;11(6):1468-1489.
10. Benjamini Y, Hochberg Y. Controlling the False Discovery Rate: A Practical and Powerful Approach to Multiple Testing. Journal of the Royal Statistical Society Series B (Methodological). 1995;57(1):289-300.

**Supplementary Tables**

Supplementary Table S1 Clinical and pathological characteristics between low CD47 expression with high CD47 expression DLBCL patients according to RNA sequencing expression

| Characteristic | Overall (n=702) n (%) | Low CD47 expression (n=242) n (%) | High CD47 expression (n=460) n (%) | P value |
| --- | --- | --- | --- | --- |
| Age, y |  |  |  |  |
| Median (IQR) | 61 (17-92) | 60 (18-90) | 61 (17-92) |  |
| ≤60 | 336 (47.9) | 123 (50.8) | 213 (46.3) | 0.2543 |
| ＞60 | 366 (52.1) | 119 (49.2) | 247 (53.7) |  |
| Gender |  |  |  |  |
| Male | 386 (55.0) | 142 (58.7) | 244 (53.0) | 0.1538 |
| Female | 316 (45.0) | 100 (41.3) | 216 (47.0) |  |
| IPI risk group |  |  |  |  |
| 0-2 | 386 (55.0) | 133 (55.0) | 253 (55.0) | 0.9917 |
| 3-5 | 316 (45.0) | 109 (45.0) | 207 (45.0) |  |
| ECOG |  |  |  |  |
| <2 | 621 (88.5) | 214 (88.4) | 407 (88.5) | 0.9847 |
| ≥2 | 81 (11.5) | 28 (11.6) | 53 (11.2) |  |
| Ann Arbor stage |  |  |  |  |
| I-II | 279 (39.7) | 94 (38.8) | 185 (40.2) | 0.7236 |
| III-IV | 423 (60.3) | 148 (61.2) | 275 (59.8) |  |
| Extranodal sites |  |  |  |  |
| <2 | 447 (63.7) | 151 (62.4) | 296 (64.3) | 0.6094 |
| ≥2 | 255 36.3) | 91 (37.6) | 164 (35.7) |  |
| LDH |  |  |  |  |
| ≤normal | 264 (37.6) | 103 (42.6) | 161 (35.0) | **0.0493** |
| ＞normal | 438 (62.4) | 139 (57.4) | 299 (65.0) |  |
| First-line therapy |  |  |  |  |
| R-CHOP | 436 (62.1) | 145 (59.9) | 291 (63.3) | 0.3854 |
| Others | 266 (37.9) | 97 (40.1) | 169 (36.7) |  |
| First-line therapeutic effect |  |  |  |  |
| CR/PR | 538 (76.6) | 190 (78.5) | 348 (75.7) | 0.6036 |
| SD/PD | 151 (21.6) | 47 (19.4) | 104 (22.6) |  |
| NR | 13 (1.8) | 5 (2.1) | 8 (1.7) |  |
| Cell of origin (n=620) |  |  |  |  |
| GCB | 238 (38.4) | 99 (47.1) | 139 (33.9) | **0.0013** |
| Non-GCB | 382 (61.6) | 111 (52.9) | 271 (66.1) |  |
| DH (n=615) | 22 (3.6) | 12/222 (5.4) | 10/393 (2.5) | 0.0665 |
| DE (n=640) | 166 (25.9) | 59/219 (26.9) | 107/421 (25.4) | 0.6762 |
| Abbreviations: IQR, interquartile range; ECOG, eastern cooperative oncology group; LDH, lactate dehydrogenase; R-CHOP, cyclophosphamide, doxorubicin, vincristine, and prednisone; CR/PR, complete/partial remission; SD/PD, stable/progressive disease; GCB, germinal center B-cell; DH, double hit; DE, double expression | | | | |
|  |  |  |  |  |
|  |  |  |  |  |
|  |  |  |  |  |
|  |  |  |  |  |

**Supplementary Figures**

Supplementary Figure S1 Flowchart of the patient selection and methods

Supplementary Figure S2

1. mRNA Expression of SIPRα in patients with high CD47 expression (n=460) and low CD47 expression (n=242).
2. mRNA Expression of TSP1 in patients with high CD47 expression (n=460) and low CD47 expression (n=242).


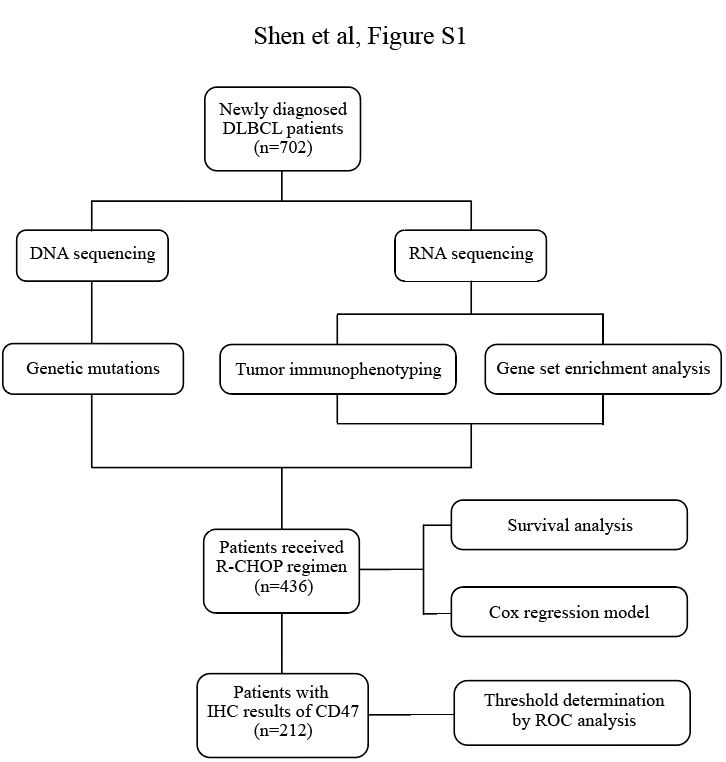


Abbreviations: DLBCL, diffuse large B-cell lymphoma; R-CHOP, rituximab, cyclophosphamide, doxorubicin, vincristine, and prednisone; IHC, immunohistochemistry; ROC, receiver operator characteristic.


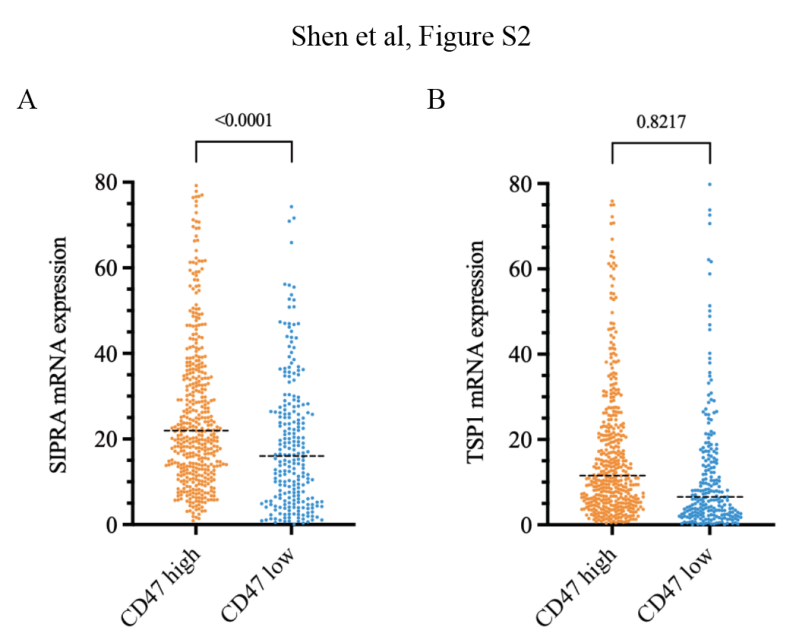

Supplement: Supplementary file 1 — Supporting Information [file CTM2-14-e1532-s001.docx]
